# Enhancing Osteoporosis Management: A Thorough Examination of Surgical Techniques and Their Effects on Patient Outcomes

**DOI:** 10.7759/cureus.59681

**Published:** 2024-05-05

**Authors:** Mihnea Popa, Adrian Cursaru, Bogdan Cretu, Sergiu Iordache, Georgian L Iacobescu, Razvan Spiridonica, Bogdan Serban, Catalin Cirstoiu

**Affiliations:** 1 Orthopedics and Traumatology Department, University Emergency Hospital, Bucharest, ROU; 2 Orthopaedics and Trauma, University of Medicine and Pharmacy Carol Davila, Bucharest, ROU

**Keywords:** femoral fractures, elderly patients, open reduction and internal fixation, osteoporosis, risk factors

## Abstract

Managing osteoporotic fractures in older individuals is a difficult task in orthopedic surgery. It requires a careful approach that combines advanced diagnostic methods, customized surgical treatments, and comprehensive rehabilitation strategies. This article presents the results of an analysis carried out at the University Emergency Hospital, Bucharest. The analysis specifically examines the treatment of osteoporotic fractures using different osteosynthesis techniques. Although diagnostic tools like dual-energy X-ray absorptiometry (DXA) and Fracture Risk Assessment Tool (FRAX) have improved, a considerable number of fractures still happen in people who do not have obvious osteoporosis. This emphasizes the importance of using additional diagnostic measures such as high-resolution peripheral quantitative computed tomography (HR-pQCT) and quantitative computed tomography (QCT) to improve the accuracy of predictions. The study demonstrates the intricate nature of surgical decision-making and the significance of adjusting techniques to meet the specific needs of each patient. An instance of osteosynthesis failure resulting from the inappropriate choice of method highlighted the crucial significance of a thorough preoperative assessment. The discussion highlights the importance of early mobilization and rehabilitation in reducing the risks associated with prolonged immobilization and improving patient recovery. This paper strongly supports the use of evidence-based and patient-centered methods in the management of osteoporotic fractures. It emphasizes the importance of utilizing the most recent advancements in diagnostic and surgical technologies. Promising advancements in orthopedic medicine lie in the future, particularly in the integration of interdisciplinary research and personalized medicine. These advancements have the potential to enhance patient outcomes in this population that is at high risk.

## Introduction

The management of fractures in patients with osteoporosis is a significant challenge and a critically important area in the evolving field of orthopedic medicine. Osteoporosis is a condition that affects the entire body and is characterized by a decrease in bone density, deterioration of the structure of the bones, and an increased likelihood of fractures, especially in the hip, spine, and forearm. These fractures not only lead to higher rates of illness and death but also create a substantial economic burden. The intricacy of managing osteoporosis is exacerbated by the constraints of existing diagnostic tools and the variability in fracture risk among diverse populations [1-3].

Dual-energy X-ray absorptiometry (DXA) and the Fracture Risk Assessment Tool (FRAX) are fundamental techniques used to evaluate the risk of fractures. They involve measuring the density of bone in a specific area and considering different clinical factors that contribute to the risk. Nevertheless, most fractures caused by osteoporosis happen in people whose bone mineral density (BMD) is above the threshold for osteoporosis and who do not have a high FRAX score [3,4]. This emphasizes the necessity for further diagnostic methods. Researchers have been investigating the relationship between cortical bone traits, which make up 80% of the skeleton, and the risk of fractures. This has prompted the use of high-resolution peripheral quantitative computed tomography (HR-pQCT) and quantitative computed tomography (QCT) as tools to improve the accuracy of current prediction methods [1,4,5].

Managing osteoporotic fractures, especially hip fractures, is difficult due to high mortality rates, substantial morbidity, and a significant impact on functional status. Although there have been improvements in our comprehension and management of osteoporosis, there are still significant deficiencies in the distribution of knowledge and strategies for preventing fractures [3,5]. This is supported by the fact that only 30-40% of patients regain their previous level of functioning after experiencing a hip fracture caused by osteoporosis. Additionally, there is a cumulative mortality rate of 20-40% within the first year following the fracture. These statistics highlight the pressing necessity for enhanced management strategies and interventions [4,6,7].

This article seeks to offer a current comprehensive review on the occurrence, development, identification, and treatment of osteoporosis and related fractures. This work aims to improve patient outcomes by conducting a thorough review of the most recent evidence, with a focus on addressing the unmet goals of fracture prevention. Adding extra bone measurements, like trabecular bone score (TBS), and taking into account population-specific data and treatment thresholds are important actions to improve the accuracy of fracture risk assessments. Moreover, the investigation of clinical risk factors and the creation of customized management strategies are crucial for advancing the treatment of patients with osteoporosis [1,8-10].

When examining the intricacies of managing osteoporosis, it is crucial to acknowledge the multifaceted characteristics of this disease and the constraints of current methods for diagnosing and treating it. This article aims to contribute to the ongoing discussion in the field of orthopedic medicine by using a collaborative and evidence-based approach. The ultimate objective is to enhance the quality of life for patients who are experiencing the debilitating consequences of osteoporosis [7,11].

## Materials and methods

This retrospective analysis was carried out at the Orthopedics and Traumatology Clinic of the University Emergency Hospital Bucharest (SUUB), Bucharest, Romania. The study focused on the treatment methods used for fractures in patients who have been diagnosed with osteoporosis. The study duration spanned from February 2023 to February 2024, and it received approval from the Ethics Council of the Emergency University Hospital Bucharest.

The study encompassed a group of patients treated for fractures that were identified as osteoporosis-related. These fractures were determined to be caused by osteoporosis based on radiologic evidence from plain radiographs and the nature of the incidents, typically involving low-energy trauma The focus of the study was on patients who received different osteosynthesis techniques based on the location of the fracture and their individual needs. The selection criteria were formulated to encompass a wide range of osteoporotic fracture cases, thus offering valuable insights into the difficulties and results linked to various osteosynthesis techniques in this group of patients.

The study included patients of both sexes and a wide range of ages, which accurately reflected the demographic diversity commonly observed in fractures related to osteoporosis. The surgical procedures were carried out by a group of experienced orthopedic and traumatology surgeons at SUUB, guaranteeing a superior level of medical attention.

The study documented the osteosynthesis techniques applied, including open reduction and internal fixation (ORIF) with locking compression plates (LCPs), intramedullary nailing, and percutaneous pinning. These surgical approaches and the choice of anesthesia (general or spinal) were tailored to each patient's specific needs, considering factors such as fracture location, severity of osteoporosis, overall health, and personal preferences. A standardized postoperative care protocol was implemented to ensure uniformity in patient treatment and recovery, encompassing pain management, mobilization, and rehabilitation. The rehabilitation program commenced on the first day after the operation, with an initial emphasis on exercises to improve mobility. Over time, the program gradually advanced to include weight-bearing activities, as long as the patient could tolerate them.

Fractures were categorized based on their anatomical position and the particular technique of bone fixation employed. The assessment of clinical outcomes involved the utilization of appropriate scoring systems specifically designed for the type of fracture, such as the Harris Hip Score (HHS) for hip fractures. Radiographic evaluations were performed to assess the progress of fracture healing, the stability of the implant, and the presence of osteointegration or any complications.

## Results

We conducted a retrospective analysis on nine elderly patients who received surgical treatment for different types of fractures, with a specific emphasis on cases complicated by osteoporosis. These cases underwent treatment at the Orthopedics and Traumatology Clinic of the SUUB, showcasing the various difficulties and results linked to surgical intervention in this specific population. One notable instance of osteosynthesis failure resulting from an improper method selection was observed, highlighting the crucial significance of customized treatment strategies.

Case 1

The treatment of comminuted fractures, especially in elderly patients with osteoporosis, is a complex task in orthopedic surgery. It requires both surgical accuracy and a comprehensive knowledge of bone biology and the process of healing. Case 1 illustrates a situation where a 74-year-old patient suffered a fall from a standing position. This type of incident is frequent among this age group and can result in serious injuries and reduced quality of life.

Upon arrival at the emergency department, the patient described experiencing sudden and severe pain, as well as an inability to perform normal activities. These symptoms are characteristic of a serious skeletal injury. The radiological assessment showed a fragmented fracture at the lower end of the radius, affecting the joint. These fractures are identified by the presence of several bone fragments. When they occur near a joint, they increase the chances of developing post-traumatic osteoarthritis, which makes the patient's recovery and functional prognosis more challenging.

Considering the fragmented nature of the fracture and the patient's advanced age, which is frequently associated with reduced bone density caused by osteoporosis, the choice to proceed with surgical treatment was made deliberately and decisively. The reasoning was clear: orthopedic, non-surgical treatment was considered insufficient and likely to lead to less-than-ideal results, such as failure to heal, improper healing, or long-lasting functional limitations. On the other hand, surgical intervention, which focuses on achieving precise alignment and secure attachment, provides the opportunity to restore joint functionality and reduce the likelihood of future complications.

**Figure 1:**
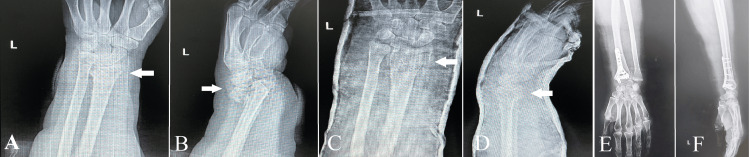
Case 1 - arrows point to the comminuted fracture This image displays the radiographs of a patient upon their arrival in the emergency room, revealing a severely fragmented fracture at the distal radial epiphysis (images A and B). An orthopedic reduction procedure was carried out, resulting in a satisfactory alignment considering the complexity of the fracture (as shown in images C and D). Due to the patient's advanced age and the low quality of their bones, osteosynthesis was performed to stabilize the fracture (images E and F), showcasing a strategic approach to handling difficult cases like this.

This case highlights the crucial significance of a customized, patient-focused strategy in the treatment of intricate fractures in older individuals (Figure 1). The objective was to enhance the patient's recovery process and enable them to regain their previous level of activity and quality of life through careful surgical planning and execution. This underscores the delicate equilibrium between technical expertise and clinical decision-making in orthopedic surgery.

Case 2

Case 2 provides a clear example of the intricate difficulties and obstacles involved in treating comminuted fractures of the proximal humerus, specifically in older individuals. This case pertained to a 68-year-old individual who suffered a fall from a standing position. As a result, the patient experienced intense pain and limited functionality in the shoulder area. This injury not only affects the physical integrity of the skeletal structure but also greatly hinders the patient's ability to carry out daily tasks and live independently.

Upon reaching the emergency department, the patient's clinical condition, marked by sudden and severe pain and a noticeable reduction in shoulder movement, required urgent evaluation using radiological imaging. The imaging studies confirmed a complex fracture of the upper arm bone near the shoulder, which also affects the joint. Fractures in this area pose a significant challenge because the bone near the joint is fragmented, making surgical reconstruction and the restoration of normal anatomy and function more difficult.

Managing comminuted proximal humerus fractures in elderly patients necessitates a nuanced strategy that carefully considers the objectives of attaining mechanical stability while maintaining the joint's functionality. Considering the patient's age, it is probable that the presence of osteoporosis played a role in the complexity of the fracture, which in turn made the surgical intervention more challenging. The decision to proceed with surgery in this case was based on the recognition that non-surgical treatment options would probably lead to unsatisfactory results, including prolonged pain, reduced shoulder function, and potentially, the emergence of post-traumatic osteoarthritis.

**Figure 2:**
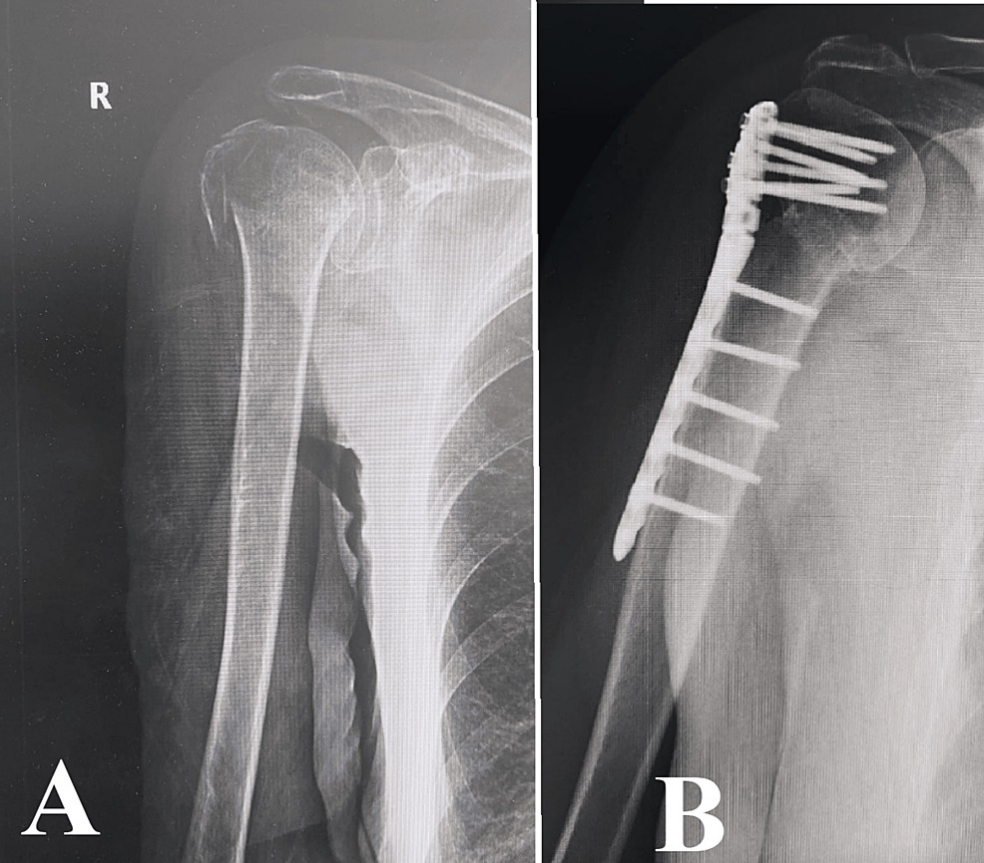
Case 2 Image A displays a humeral head fracture characterized by fractures at the anatomical neck, surgical neck, and greater tubercle. Image B showcases the precise anatomical osteosynthesis accomplished through the use of a locking plate. It demonstrates the meticulous method employed to restore both the structural integrity and functionality in intricate shoulder injuries.

This case highlights the crucial significance of using a comprehensive, interdisciplinary approach to manage complicated shoulder fractures in older individuals (Figure 2). The surgical procedure is designed to restore the anatomy of the proximal humerus as accurately as possible, taking into account the specific characteristics of the fracture and the patient's overall health status. The objective is to optimize the patient's functional recovery and quality of life by employing careful preoperative planning, precise surgical technique, and a dedicated postoperative rehabilitation program. This approach emphasizes the complex relationship between surgical expertise, patient-centered care, and the management of osteoporotic fractures.

Case 3

Case 3 explores the complex field of orthopedic trauma, describing a compelling situation where an 88-year-old patient arrived at the emergency department with severe pain and significant loss of shoulder function after falling from a standing position. This case exemplifies the complex difficulties involved in managing severe skeletal injuries in elderly individuals, especially when combined with the complexities of osteoporosis and the increased likelihood of complications.

The radiological assessment showed a complex fracture that starts from the upper arm bone and extends into the upper part of the bone shaft, indicating the seriousness of the injury and the complex interaction of forces during the trauma. The presence of both the joint and the metaphyseal-diaphyseal region greatly complicates the treatment plan, requiring a surgical approach that addresses the requirements for joint stability and the restoration of the humeral shaft's integrity.

Given the specific characteristics of the fracture and the patient's physical condition, the use of a centromedullary rod along with cerclage wiring is a highly advanced surgical approach. The centromedullary rod provides internal stabilization for the fracture, acting as a framework for bone healing. The cerclage wiring helps secure the broken bone fragments, ensuring proper alignment and promoting the restoration of the bone's structural integrity.

This case emphasizes the crucial significance of employing a strategic and personalized method for surgical treatment in elderly patients with intricate fractures. The choice to utilize both a centromedullary rod and cerclage wiring exemplifies the intricate decision-making involved in modern orthopedic trauma surgery. The statement demonstrates a profound comprehension of the biomechanical principles that govern fracture treatment, the physiological difficulties associated with aging and osteoporosis, and the primary objective of enhancing function and quality of life for this susceptible group of patients.

**Figure 3:**
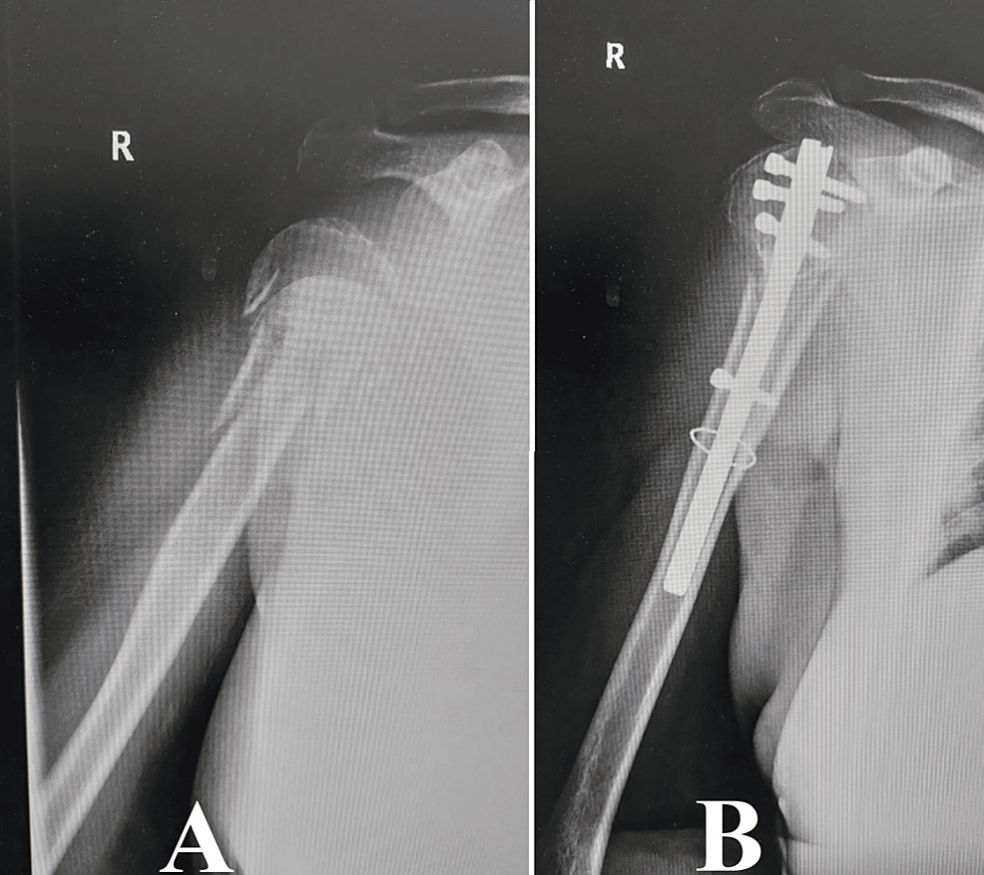
Case 3 Image A displays a fracture in the humeral head that extends into the proximal diaphysis. Image B displays the use of a centromedullary nail that is locked at both ends of the fracture to perform osteosynthesis. Additionally, a cerclage wire is used to stabilize the fracture site. This approach emphasizes the incorporation of internal fixation methods to attain ideal alignment and stability in intricate fractures of the upper limb.

This approach demonstrates a dedication to improving patient outcomes in the treatment of difficult orthopedic problems in older adults. It emphasizes the importance of innovation, precision, and patient-centered care in managing fractures in the elderly (Figure 3).

Cases 4 and 5

Cases 4 and 5 shed light on the complex range of orthopedic treatment for elderly patients, showcasing two specific cases of trochanteric fractures in individuals aged 78 and 84 years, respectively. These cases exemplify the difficulties and factors involved in treating older patients, who frequently have multiple medical conditions and weakened bone structure caused by osteoporosis. The fractures, which are identified by their minimal displacement and fragmentation, provide a valuable understanding of the decision-making involved in using dynamic hip screw (DHS) systems for surgical intervention.

The patients in question experienced fractures in the trochanteric region, which were anatomically reduced. However, due to the age of the patients, a surgical approach was necessary to not only address the immediate structural integrity of the hip but also take into account the wider implications of postoperative recovery and rehabilitation. The selection of DHS as the fixation method highlights a strategic preference for stabilization techniques that promote early mobilization and weight-bearing. Ensuring mobility is especially crucial in geriatric care, as prolonged immobility can trigger a series of complications, such as deep vein thrombosis, pulmonary embolism, muscle atrophy, and further deterioration in functional status.

The DHS system is highly regarded for its capacity to offer secure fixation while enabling controlled compression at the site of the fracture during movement. This makes it an ideal choice for these types of clinical situations. The DHS system promotes early weight-bearing after surgery, which helps maintain muscle tone and joint function. This leads to a faster and more complete recovery. This approach demonstrates a comprehensive comprehension of the biomechanical principles involved in managing fractures. Additionally, it embodies a patient-centered philosophy that places importance on functional outcomes and quality of life.

**Figure 4:**
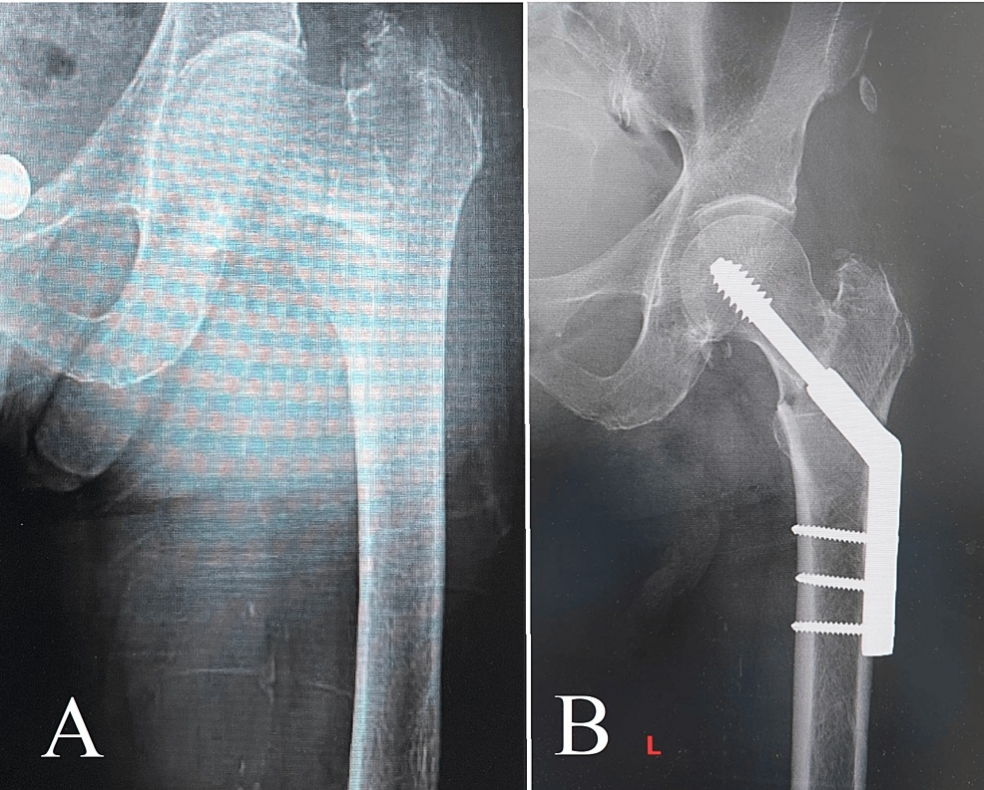
Case 4 Image A displays an intertrochanteric fracture, which is a frequently encountered but complex injury that necessitates meticulous treatment. Image B demonstrates the successful anatomical realignment achieved through the use of a dynamic hip screw (DHS), highlighting the efficacy of this technique in restoring stability and function to the hip, especially in cases of fractures involving the upper part of the thigh bone.

**Figure 5:**
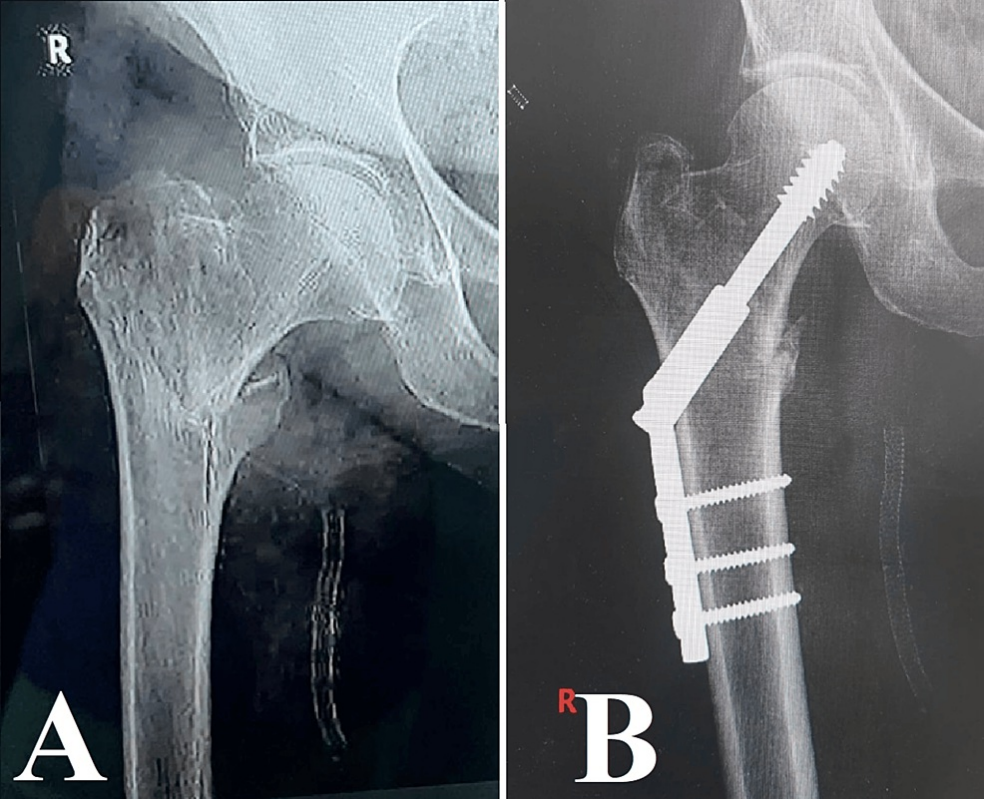
Case 5 Image A displays an intertrochanteric fracture, which is a frequently encountered but complex injury that necessitates meticulous treatment. Image B demonstrates the successful anatomical realignment achieved through the use of a dynamic hip screw (DHS).

When analyzing the clinical narratives of these two cases (Figures 4, 5), it becomes clear that effectively treating trochanteric mass fractures in older individuals goes beyond simply performing surgical techniques with skill. The approach involves a comprehensive plan that combines surgical proficiency, knowledge of the physiological changes that occur in older adults, and a dedication to enhancing the recovery process after surgery. By using DHS fixation in a careful and thoughtful way, these cases provide valuable knowledge about the changing approaches in orthopedic surgery. They emphasize the significance of customized treatments that meet the specific requirements of elderly patients, while also working toward the main objective of improving mobility, independence, and overall health.

Cases 6 and 7

Cases 6 and 7 explore the complex field of orthopedic trauma, focusing on the difficulties faced in treating trochanteric mass fractures with extensive fragmentation and metaphyseal extension in 81- and 82-year-old patients, respectively. These cases are especially enlightening, as they highlight the intricacies of treating high-risk fractures in older adults, where maintaining mobility and independence is crucial, despite the compromised ability for bone healing.

The fractures observed in these cases were marked by important comminution, which resulted in the fragmentation of the peritrochanteric region as well as the subtrochanteric cortex. This component is crucial for the stability of any method used to fix the fracture. This situation posed an increased risk of mechanical failure of the surgical assembly, requiring a meticulous approach to surgical intervention.

The choice to employ the Gamma nail system in these cases demonstrates a strategic adjustment to the distinct difficulties presented by the fracture patterns and the elderly patients' advanced age. The Gamma nail is an intramedullary fixation device that is specifically engineered to offer stable support for complicated proximal femoral fractures. It enables early mobilization while minimizing the risk of structural deterioration when subjected to normal physiological loads.

Due to the significant fragmentation and weakened structural strength of the outer layer of the thigh bone, a careful post-surgery rehabilitation plan was required. Patients were instructed to ambulate without putting any weight on the operated limb for a duration of four weeks following the surgery. The objective of this approach was to achieve a careful equilibrium between encouraging early movement of the joints and activation of the muscles. These factors are important in preventing the harmful consequences of prolonged immobilization. Additionally, the approach aimed to maintain the stability of the surgical fixation until the femur had healed enough to withstand the forces exerted during weight-bearing activities.

**Figure 6:**
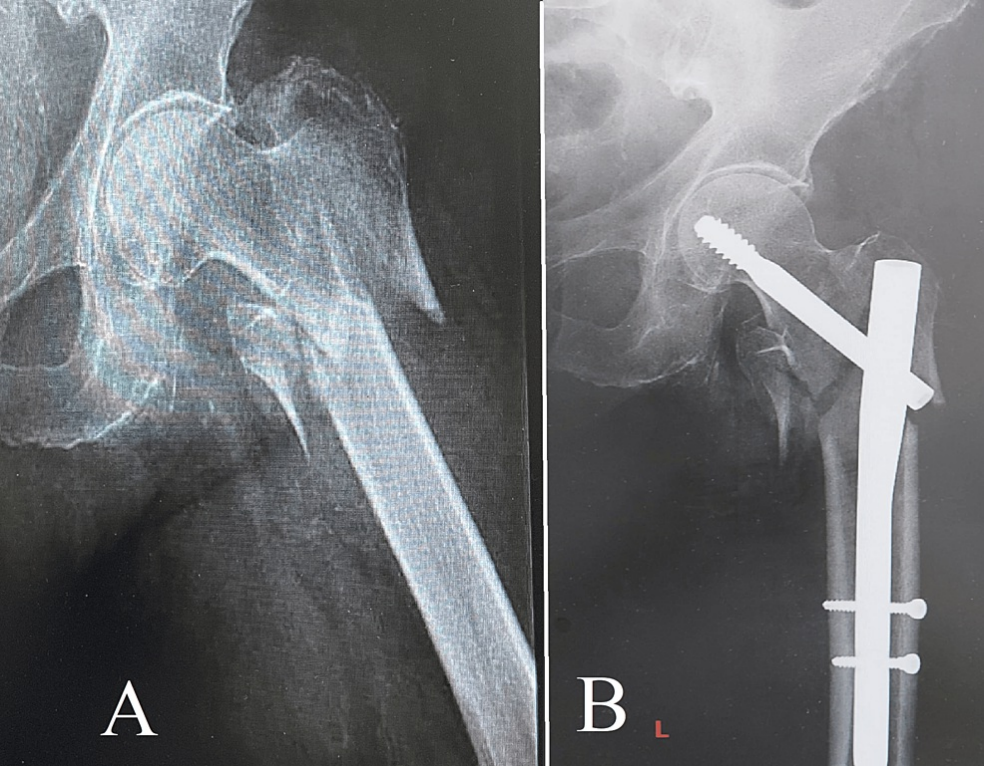
Case 6 Image A displays a comminuted fracture of the trochanteric mass, along with a transverse subtrochanteric fracture. This complex injury presents significant treatment challenges. Image B demonstrates the successful reduction and fixation achieved with a Gamma nail, showcasing the effectiveness of this intramedullary fixation device in treating complex fractures. The application of the Gamma nail guarantees stabilization at the fracture sites, promoting ideal conditions for healing and assisting the patient's recovery process.

**Figure 7:**
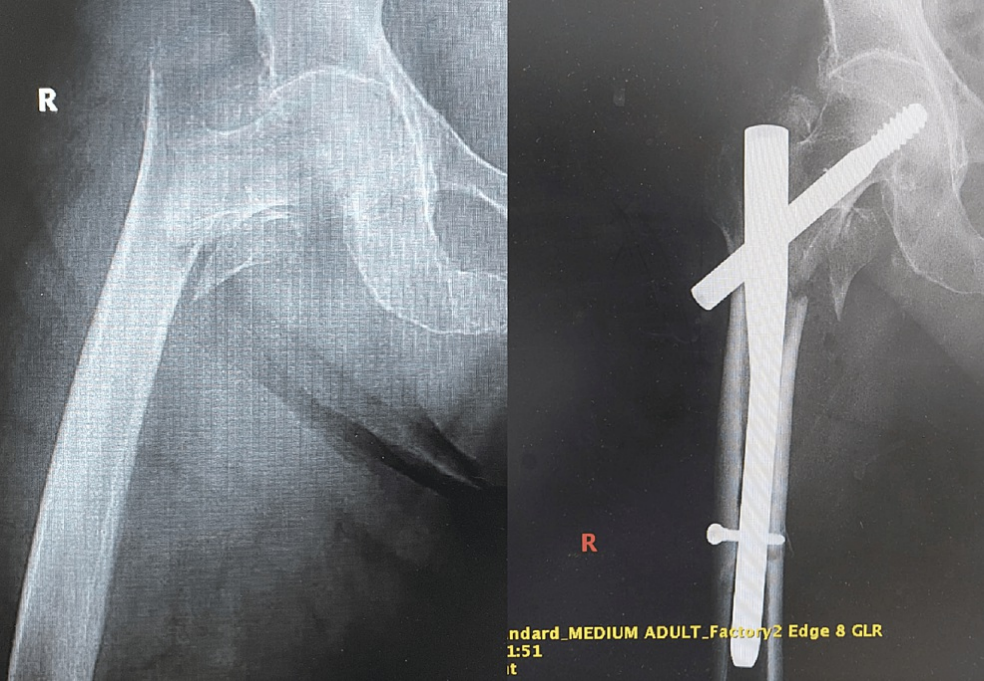
Case 7 Image A shows a severely fragmented fracture of the trochanteric mass with nearly total destruction, which poses a very difficult situation for orthopedic treatment. Image B shows the utilization of a Gamma nail to stabilize the fracture. The selection highlights the appropriateness of the Gamma nail for ensuring secure internal fixation in situations where the remaining bone structure is severely compromised. The use of a dynamic hip screw (DHS) system at this point would have increased the risk of further compromising the strength of the remaining bone, emphasizing the importance of careful decision-making when dealing with severe fractures.

These cases (Figures 6, 7) exemplify the subtle factors that form the basis for the management of intricate fractures in geriatric patients. The utilization of the Gamma nail system, in conjunction with a customized rehabilitation protocol, demonstrates the merging of surgical advancement, biomechanical principles, and geriatric care in orthopedic practice. These cases provide valuable insights into the evolving strategies for optimizing outcomes in managing fractures in elderly patients. They highlight the importance of individualized treatment plans that consider the unique physiological and functional needs of older patients while balancing mechanical stability and biological healing.

Case 8

Case 8 presents an 87-year-old patient who experienced a hip injury resulting in a fracture of the femoral neck. This case exemplifies the crucial choices that need to be made when treating hip fractures in older individuals. The potential for complications after the injury, such as aseptic necrosis of the femoral head, requires a careful and sophisticated surgical approach.

In this case, the decision to perform a hip hemiarthroplasty was based on a thorough assessment of the patient's clinical condition, taking into account the potential risk of reduced blood flow to the femoral head after a femoral neck fracture. Aseptic necrosis, also known as avascular necrosis, is a serious complication caused by the interruption of blood supply to the femoral head, leading to the death of bone tissue. This ultimately causes joint dysfunction and intense pain. In older individuals, who have limited physiological capacity for regeneration, the consequences of necrosis are especially severe, often resulting in a significant decrease in mobility and quality of life.

Hip hemiarthroplasty is a surgical procedure that involves replacing the femoral head and neck with a prosthetic implant while keeping the natural acetabulum intact. It is considered a strategic solution in this situation. This procedure not only treats the immediate structural damage caused by the fracture but also prevents the risk of aseptic necrosis by removing the need for the compromised blood supply to the femoral head. In addition, hemiarthroplasty promotes early mobilization, which is crucial for the recovery of elderly patients. This is achieved by promptly stabilizing the hip joint and minimizing the time required for postoperative immobilization.

**Figure 8:**
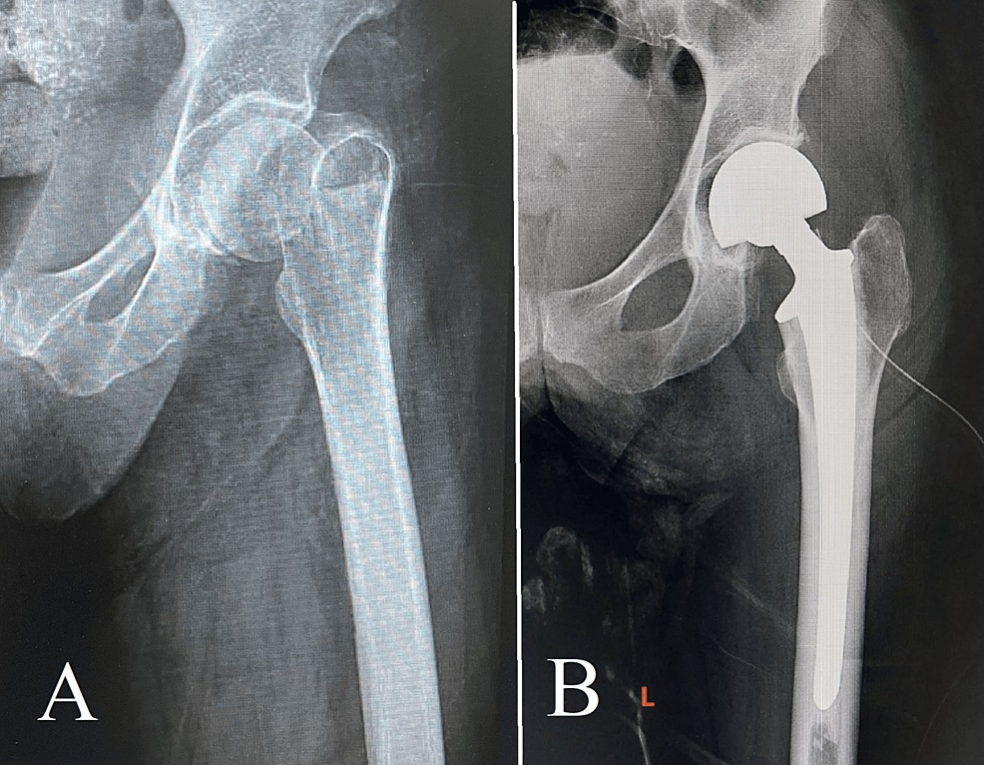
Case 8 Image A depicts a typical femoral neck fracture with associated vascular disruption, which poses a significant challenge due to the potential for compromised blood flow to the femoral head. The choice to undergo a bipolar hemiarthroplasty, as depicted in image B, demonstrates a deliberate strategy to maintain joint functionality while also addressing the risk of avascular necrosis.

This case highlights the complex relationship between making surgical decisions and the overall goals of maintaining function, reducing complications, and improving recovery after hip fractures in older individuals (Figure 8). The decision to perform hip hemiarthroplasty is based on careful clinical assessment, taking into account the underlying causes of femoral neck fractures, the potential risks of different treatment options, and the need to customize interventions to the patient's overall health and functional objectives. Orthopedic surgery is constantly pushing the boundaries of geriatric fracture management by using personalized care strategies. This ensures that elderly patients receive the best possible treatment outcomes, which in turn helps them maintain their mobility and quality of life.

Case 9

This case pertained to a fracture of the femoral stem, specifically classified as a Vancouver B fracture, near an existing hip prosthesis. The patient's specific condition was initially treated with a method that was considered unsuitable. This decision was influenced by an underestimation of the fragility of the osteoporotic bone and the stability of the implant. As a result, the outcome was suboptimal.

Complications arose due to the selected osteosynthesis method, resulting in non-union and subsequent failure of the implant. As a result, revision surgery was required. This case emphasized the intricacies of handling fractures in bones with osteoporosis, particularly when there are prosthetic implants involved. It underscored the critical significance of choosing the appropriate method based on a comprehensive evaluation of bone strength and implant stability.

Case 9 eloquently demonstrates the intricacies and difficulties involved in managing fractures in elderly patients, specifically in the case of a 91-year-old patient with a highly fragmented fracture of the entire trochanteric massif. This situation highlights the crucial significance of choosing a suitable surgical procedure that matches the biomechanical requirements of the fracture and the physiological characteristics of the elderly patient group.

The choice to employ a DHS in this particular case was based on its extensive use and proven effectiveness in treating trochanteric fractures. The DHS system is specifically engineered to facilitate controlled compression at the fracture site, thereby promoting healing through the enhancement of bone contact and stability. Nevertheless, the severe fragmentation and lack of stability in the patient's fracture posed a unique challenge, as it made the DHS less effective than expected. The postoperative medialization of the fracture site revealed the shortcomings of DHS in treating fractures with extensive fragmentation and compromised structural stability.

Upon careful consideration of the surgical outcome, it is clear that the use of a Gamma nail, which is an intramedullary fixation device, could have resulted in a more positive prognosis in this specific case. The Gamma nail is specifically designed to offer secure internal fixation for fractures in the proximal femur, and it is particularly effective in managing the intricate biomechanical challenges presented by comminuted trochanteric fractures. The intramedullary positioning of the device allows for a more precise alignment with the mechanical axis of the bone, resulting in improved stability and potentially reducing the risk of the fracture becoming misaligned.

**Figure 9:**
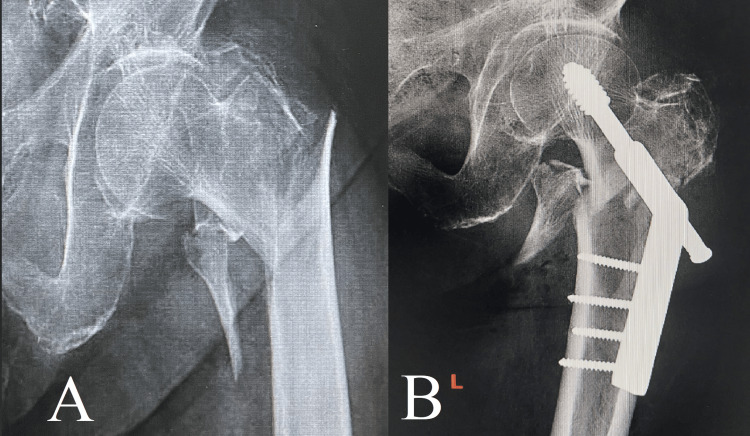
Case 9 Image A displays a comminuted fracture of the trochanteric mass, which is comparable to the previously discussed case 7. At this point, the decision was made to use a dynamic hip screw (DHS), as shown in image B. Nevertheless, it seems that this decision may not have been the most appropriate. The DHS, although efficacious for specific fracture types, may not offer the requisite stability or facilitate ideal healing in instances of severe comminution, as demonstrated by the difficulties encountered in this particular situation. This scenario emphasizes the significance of thoroughly assessing the distinct attributes of each fracture and contemplating alternative methods of fixation that may be more suitable for addressing the complexities associated with highly comminuted injuries.

This case highlights the intricate decision-making process necessary for the surgical treatment of complicated fractures in elderly patients (Figure 9). It emphasizes the importance of conducting a comprehensive preoperative evaluation that takes into account the unique features of the fracture, the mechanical properties of the fixation devices, and the patient's overall health condition. Furthermore, it emphasizes the significance of being able to adjust surgical planning to align with the changing understanding of fracture mechanics and patient-specific factors. This includes selecting appropriate fixation methods that can adapt to these factors. By employing a thorough and knowledgeable method, orthopedic surgeons can improve treatment plans to maximize results for elderly patients dealing with complex comminuted trochanteric fractures.

The failure observed in case 9 highlights the complex nature of treating fractures in osteoporotic patients, especially those with pre-existing prosthetic implants. The initial surgical approach did not consider the weakened bone quality and the unique factors introduced by the implant. This case highlights the importance of conducting a thorough preoperative evaluation, which should include detailed radiographic assessments and taking into account the patient's overall health and bone condition. This evaluation is crucial in determining the most suitable method for osteosynthesis.

## Discussion

Managing osteoporotic fractures, especially in older individuals, is a complex task in orthopedic surgery that requires a detailed comprehension of the disease's pathophysiology and the biomechanical principles involved in fracture treatment. This discussion combines the results of a retrospective analysis carried out at the University Emergency Hospital Bucharest with information from current literature to clarify strategies that improve patient outcomes in this vulnerable group.

Osteoporosis, a condition marked by reduced bone density and structural degradation, greatly increases the likelihood of fractures, particularly in the hip, spine, and forearm. These fractures not only worsen illness and death rates but also place a significant financial burden, highlighting the need for efficient management strategies [1-3]. Although diagnostic methodologies like DXA and FRAX have improved, a significant number of fractures still happen in people who do not have obvious osteoporosis or high FRAX scores. This shows that current predictive tools have limitations and there is a need for more diagnostic measures [3,4,12].

The integration of HR-pQCT and QCT into the diagnostic toolkit is a major advancement in improving the accuracy of fracture risk assessments. These modalities provide in-depth information about cortical bone characteristics, which make up 80% of the skeleton. As a result, they offer a more nuanced comprehension of the likelihood of fractures [1,5,6,13].

The analysis of nine cases of osteoporotic fractures treated at SUUB highlights the crucial significance of customizing surgical interventions according to the specific needs of each patient. The wide range of osteosynthesis techniques used, including ORIF with LCPs and intramedullary nailing, highlights the intricate decision-making process involved in surgically treating osteoporotic fractures. The complexity of the situation is highlighted by a case where osteosynthesis failed because an inappropriate method was chosen. This emphasizes the need for a careful evaluation before surgery, taking into account the characteristics of the fracture, the patient's overall health, and the specific difficulties presented by osteoporotic bone (case 9).

The analysis of specific instances brings attention to several crucial factors in the treatment of osteoporotic fractures. For example, the effective management of comminuted fractures in the distal radius and proximal humerus (cases 1 and 2) demonstrates the significance of attaining anatomical alignment and secure immobilization to restore joint function and reduce the likelihood of long-term complications. The case study involving an 88-year-old patient with a complex proximal humerus fracture (case 3) highlights the importance of customizing surgical techniques to meet the specific requirements of the fracture and the patient's physiological condition. In this case, the strategic utilization of a centromedullary rod and cerclage wiring proved to be essential.

The utilization of DHS in instances of trochanteric mass fractures with minimal displacement (cases 4 and 5) and the Gamma nail in cases with substantial comminution (cases 6 and 7) demonstrates a prudent implementation of biomechanical principles to guarantee stability and facilitate the healing process. Furthermore, the choice to carry out a hip hemiarthroplasty in an 87-year-old patient with a femoral neck fracture (case 8) highlights the crucial importance of surgical treatment in preventing aseptic necrosis of the femoral head and promoting early mobilization.

The combined knowledge gained from these cases, along with the examination of the unsuccessful case, highlights the complex and diverse aspects of managing osteoporotic fractures. A comprehensive and evidence-based approach, informed by the latest advancements in diagnostic and surgical techniques, is crucial for improving patient outcomes. This involves not only improving current diagnostic tools but also creating personalized treatment strategies that consider the distinct physiological and biomechanical difficulties faced by each patient. The treatment of osteoporotic fractures necessitates both an interdisciplinary approach and a thorough understanding of the advancing field of orthopedic research and clinical practice. The incorporation of innovative diagnostic tools and surgical techniques, as emphasized in the showcased cases, provides a route toward tailored and efficient treatments for this vulnerable group of patients [5,10,14,15].

The limitations of DXA and FRAX in accurately predicting fracture risk emphasize the need to include advanced diagnostic techniques such as HR-pQCT and QCT. These technologies provide a more detailed examination of bone quality, going beyond just density, enabling a more accurate evaluation of the likelihood of fractures. Visualizing and quantifying cortical and trabecular bone traits allows for a more comprehensive comprehension of bone strength and the likelihood of fracture under stress. The use of a nuanced approach to diagnosis is essential to accurately identify patients who are truly at risk of osteoporotic fractures. This approach enables targeted interventions that have the potential to greatly impact clinical outcomes [3,14,16,17].

The surgical treatment of osteoporotic fractures, as illustrated by the cases, highlights the significance of customizing techniques to meet the individual requirements of each patient. The choice between DHS and Gamma nail, for example, demonstrates the delicate equilibrium between ensuring mechanical stability and creating a favorable biological environment for healing. Case 9, which is the failure case, specifically demonstrates the negative outcomes of choosing a method without properly considering the osteoporotic condition and the specific fracture characteristics. It is crucial to conduct a comprehensive preoperative evaluation, which includes a meticulous examination of bone quality and the mechanical stresses imposed on the fixation device [12,18,19].

An additional crucial element in the management of osteoporotic fractures is the focus on prompt mobilization and rehabilitation. Surgical procedures such as the DHS and hip hemiarthroplasty, which enable patients to bear weight on their operated limb soon after surgery, are crucial in reducing the dangers associated with extended immobilization in older patients. Early mobilization not only promotes the physical recovery process but also enhances the psychological well-being of patients, thereby improving their overall recovery experience.

The field of orthopedic medicine is constantly advancing, with ongoing research and clinical trials investigating new materials, surgical techniques, and rehabilitative protocols specifically designed for individuals with osteoporosis. The incorporation of interdisciplinary research, which combines the fields of bone biology, materials science, and biomechanics, shows potential for creating new and inventive solutions to tackle the existing difficulties in fracture management. Moreover, the possibility of customized medicine, propelled by the genetic and molecular understanding of osteoporosis, could provide novel approaches for focused prevention and treatment methods [5,20-22].

## Conclusions

The management of osteoporotic fractures in elderly individuals involves an intricate combination of accurate diagnosis, surgical proficiency, and individualized treatment. The cases presented highlight the importance of using an evidence-based, patient-centered approach that takes advantage of the most recent advancements in diagnostic and surgical technologies. By promoting a more profound comprehension of osteoporosis and its influence on the likelihood of fractures, orthopedic practitioners can more effectively navigate the difficulties associated with this condition, ultimately enhancing the quality of life for patients suffering from osteoporotic fractures. To achieve the main objective of improving patient outcomes in osteoporotic fracture management, it is crucial to prioritize the ongoing incorporation of research findings into clinical practice as the field progresses.
